# Deep-learning-based segmentation of perivascular spaces on T2-Weighted 3T magnetic resonance images

**DOI:** 10.3389/fnagi.2024.1457405

**Published:** 2024-08-29

**Authors:** Die Cai, Minmin Pan, Chenyuan Liu, Wenjie He, Xinting Ge, Jiaying Lin, Rui Li, Mengting Liu, Jun Xia

**Affiliations:** ^1^Department of Radiology, The First Affiliated Hospital of Shenzhen University, Shenzhen University, Shenzhen Second People’s Hospital, Shenzhen, Guangdong, China; ^2^School of Information Science and Engineering, Shandong Normal University, Shandong, China; ^3^Five-Year Clinical Medicine, Xiangya School of Medicine, Central South University, Changsha, Hunan, China; ^4^Department of Biomedical Engineering, Sun Yat-sen University, Shenzhen, Guangdong, China

**Keywords:** perivascular spaces, Virchow-Robin spaces, deep learning, multiscale supervised, dense nesting, 3T MR image

## Abstract

**Purpose:**

Studying perivascular spaces (PVSs) is important for understanding the pathogenesis and pathological changes of neurological disorders. Although some methods for automated segmentation of PVSs have been proposed, most of them were based on 7T MR images that were majorly acquired in healthy young people. Notably, 7T MR imaging is rarely used in clinical practice. Herein, we propose a deep-learning-based method that enables automatic segmentation of PVSs on T2-weighted 3T MR images.

**Method:**

Twenty patients with Parkinson’s disease (age range, 42–79 years) participated in this study. Specifically, we introduced a multi-scale supervised dense nested attention network designed to segment the PVSs. This model fosters progressive interactions between high-level and low-level features. Simultaneously, it utilizes multi-scale foreground content for deep supervision, aiding in refining segmentation results at various levels.

**Result:**

Our method achieved the best segmentation results compared with the four other deep-learning-based methods, achieving a dice similarity coefficient (DSC) of 0.702. The results of the visual count of the PVSs in our model correlated extremely well with the expert scoring results on the T2-weighted images (basal ganglia: rs = 0.845, *P* < 0.001; rs = 0.868, *P* < 0.001; centrum semiovale: rs = 0.845, *P* < 0.001; rs = 0.823, *P* < 0.001 for raters 1 and 2, respectively). Experimental results show that the proposed method performs well in the segmentation of PVSs.

**Conclusion:**

The proposed method can accurately segment PVSs; it will facilitate practical clinical applications and is expected to replace the method of visual counting directly on T1-weighted images or T2-weighted images.

## 1 Introduction

**FIGURE 1:**
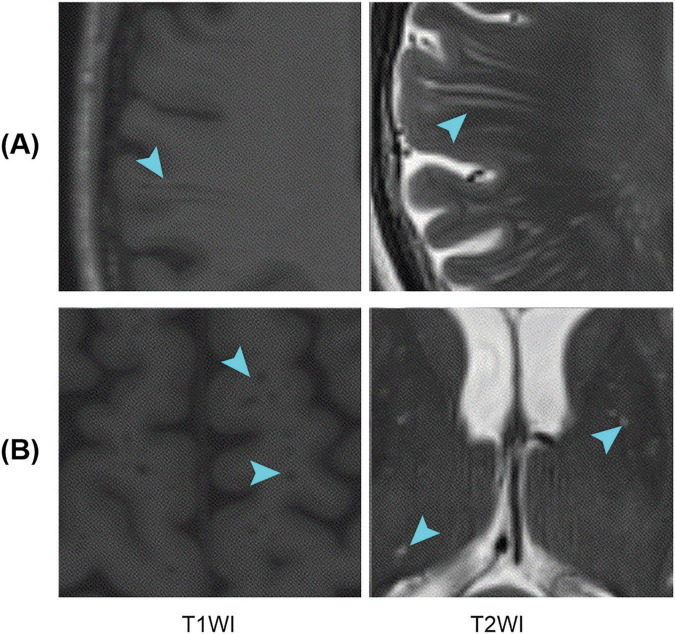
Examples of PVSs on magnetic resonance images. T1WI represents T1-weighted images, and T2WI represents T2-weighted images; **(A,B)** Indicate that the PVSs appear as lines and dots, respectively. PVSs, perivascular spaces.

Perivascular spaces (PVSs), also known as Virchow–Robin spaces, are fluid-filled spaces that surround small blood vessels in the brain and traverse through the brain substance (Ballerini et al., 2018). PVSs are primarily distributed in areas such as the white matter, basal ganglia (BG), and brainstem (Salzman et al., 2005). They play a role in cerebrospinal fluid (CSF) circulation and clearance of metabolic waste from the brain (Iliff et al., 2013; Rangroo Thrane et al., 2013; Yang et al., 2013). In magnetic resonance imaging (MRI), PVSs tend to appear as longer linear structures when the direction of penetration is parallel to the scanning plane and as small dot-like structures when they are perpendicular to the scanning plane; PVSs usually have a signal intensity similar to that of CSF (Figure 1). It is important to note that the size and number of PVSs are related to aging (Francis et al., 2019) and various diseases, including Alzheimer’s disease (AD) (Hansen et al., 2015; Boespflug et al., 2018), Parkinson’s disease (PD) (Shibata et al., 2019; Shen et al., 2021), multiple sclerosis (MS) (Wuerfel et al., 2008; Conforti et al., 2014; George et al., 2021), and small vessel disease (SVD) (Doubal et al., 2010; Zhu et al., 2010). PVS changes may reflect the pathogenesis of certain neurodegenerative diseases (Charidimou et al., 2013). Therefore, accurate identification and quantification of PVSs are crucial when studying these diseases.

Visual rating scales remain the gold standard for assessing PVSs burden, and numerous scales have been developed for this purpose (Patankar et al., 2005; Chen et al., 2011; Rowley, 2013; Wardlaw et al., 2013; Laveskog et al., 2018). Visual scoring is a simple and intuitive method; however, it only provides information about the presence and degree of PVSs. It does not offer more detailed quantitative information regarding parameters such as volume, morphological features, and distribution of features in the brain (Bouvy et al., 2016). Additionally, when using visual rating scales, scans are compared based on the category and not on the number of PVS. If the number of PVS increases over time but remains within the same category, visual scoring will not detect changes over time (Moses et al., 2023). Consequently, fully automated methods should be developed to compensate for and improve the shortcomings of the visual scoring method for rating PVSs.

In recent years, some researchers have proposed methods for automatic PVSs segmentation based on deep learning. However, most studies focused on 7T MRI (Park et al., 2016; Hou et al., 2017; Zhang et al., 2017; Lian et al., 2018; Spijkerman et al., 2022), with a relatively limited number of studies conducted on 3T MRI (Boutinaud et al., 2021; Rashid et al., 2023). Additionally, most of the study participants were healthy young people (Park et al., 2016; Hou et al., 2017; Zhang et al., 2017; Lian et al., 2018; Boutinaud et al., 2021; Spijkerman et al., 2022). Moreover, 7T MRI is rarely used in routine clinical practice. Therefore, we aimed to develop an automatic segmentation model for PVSs in T2-weighted 3T MRI based on patients with PD.

## 2 Materials and methods

### 2.1 Data

We prospectively collected cranial MR images of 20 patients with PD. The average age of the patients was 63.0 ± 10.3 years (mean ± SD, range: [42–79], median = 64 years), with a sex ratio of 1:1. As recommended by the 2023 STRIVE-2 guidelines (Duering et al., 2023), for all participants, 3D T1-weighted and high-resolution T2-weighted images were collected using a 3T MR scanner equipped with a 64-channel head coil (Magnetom Prisma; Siemens Healthineers, Germany). Detailed information on the MRI acquisition parameters is provided in Table 1.

**TABLE 1:** Magnetic resonance image acquisition parameters

	3D T1-weighted image	High-resolution T2-weighted image
TE (ms)	2.32	97
TR (ms)	1780	4010
TI (ms)	900	NA
Flip angle (°)	8	150
Field of view (mm)	240 × 240	220 × 220
Matrix size (mm)	256 × 256 × 256	320 × 320
Slice thickness (mm)	0.9	2.0
Slice gap (mm)	0	0
Scan time (min)	4.09	8.11
Voxel size (mm)	0.94 × 0.94 × 0.90	0.69 × 0.69 × 2.00
Bandwidth (Hz/Px)	200	223
Slice orientation	Sagittal	Axial

### 2.2 Production of ground truth for PVSs

To develop and assess the proposed machine learning-based method for extracting PVSs, we initially created ground truth PVSs masks through a comprehensive process involving visual inspection. An expert manually segmented the PVSs of the 20 subjects using ITK-SNAP software (version 3.8.0). These segments were then refined by two experienced neuroradiologists, each with over 5 years of clinical practice experience. The experts meticulously reviewed the preliminary ground truth that was formulated by combining the T2-weighted images. Disagreements were resolved through discussion to ensure the precision of the ground truth for effective model training.

### 2.3 Visual counting of PVSs burden

Three independent raters counted the PVSs on T2-weighted images and the model segmentation results for all participants. Among the raters, one was a senior neurologist and the other two were senior neuroradiologists, each with over 20 years of clinical practice experience. Both the senior neurologist and neuroradiologist were provided the T1-weighted and T2-weighted images for all participants. They independently counted the PVSs in selected slices on T2-weighted images. Another senior neuroradiologist counted the PVSs in the same slices as those in the model segmentation results. Slice selection: for the BG, the slice shows the anterior commissure; for the centrum semiovale CSO, the slice was 1 cm above the uppermost part of the lateral ventricles. The number of PVSs in these slices correlates well with the number of PVSs in the entire volume of the region (Adams et al., 2013).

### 2.4 Network architecture

**FIGURE 2:**
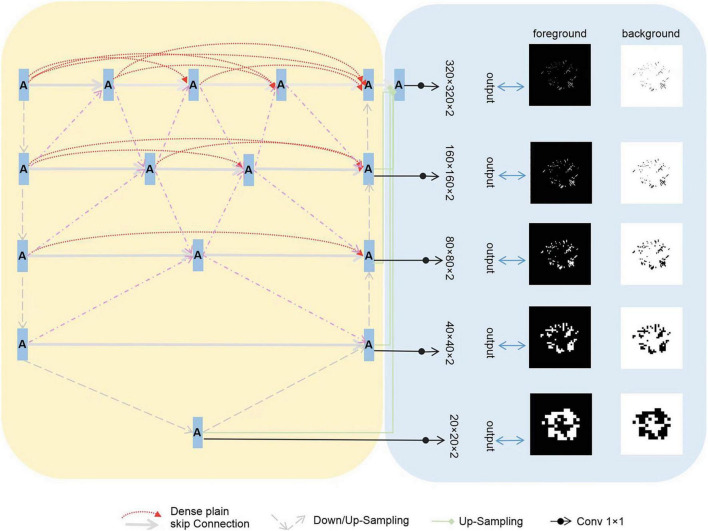
Framework of the proposed segmentation network.

We assembled a series of U-shaped sub-networks to create a densely nested structure. This design caters to the varying optimal receptive fields required for targets of different sizes, with the depth of each U-shaped subnetwork being ideal for specific target sizes. This concept was further enhanced by placing multiple nodes along the encoder-to-decoder pathways. These nodes were interconnected and formed nested networks. As depicted in Figure 2, each node processes the features of its own layer as well as those of adjacent layers, enabling a thorough multilayered fusion of features. Consequently, this architecture effectively preserves the representation of smaller targets in the deeper layers, leading to improved outcomes, and we propose the incorporation of multi-scale Highlighting Foregrounds (HFs) to enhance deep supervision within the densely nested U-Net framework. Based on the above characteristics, we named the model MfNS_De. Our modified network, depicted in Figure 2, is based on an encoder-decoder structure. In both the training and testing phases, the network processed individual slices of T2-weighted (T2w) modalities as inputs. We chose axial slices for the 2D input because of the discontinuities in the third dimension, a characteristic inherent to our images.

In our network decoder, we integrated modified label images processed using the multi-scale HF approach at intermediate layers (as shown in Figure 2, right). These intermediate output convolutional layers transform feature maps from different decoder layers into multi-scale segmentation probability maps. Concurrently, multi-scale HFs were applied to down sample the label or ground truth images to various scales (Figure 2, right). The foreground/background label images created using multi-scale HFs were then utilized to generate losses by comparison with their corresponding outputs. For the loss function, we used a soft dice score.

### 2.5 Training details

We have implemented the proposed network on an NVIDIA Tesla V100-SXM2 computer using PyTorch. To compare the model performance between our network and others, we employed five-fold cross-validation. All subjects were initially categorized into five groups, with the age distribution being fairly uniform across these groups. During each cycle of model training, one group was designated as the test set, while another one was randomly chosen as the validation set, and the remaining three groups were used as the training sets. The composition of the training, validation, and test sets remained the same in all comparison networks. The hyper-parameters of the networks are set as follows: mini-batch = 4, optimiser = Adagrad, learn rate = 0.05, epoch = 1000. Furthermore, the version of nnU-Net at the time is nnU-Net v2, which is configured as 3D full-resolution, to ensure a fair comparison. The model was trained for 1,000 epochs, and good convergence was achieved after 50 epochs. Therefore, we selected the model at epoch 50 as the well-trained model for the test data. When calculating the performance metrics (e.g., DSC, SEN, and PPV), we used the same computational method for each model to ensure the consistency and comparability of the results.

### 2.6 Evaluation metrics

Segmentation performance was evaluated using the dice similarity coefficient (DSC), sensitivity (SEN), and positive predictive value (PPV), as defined below:


$$D\,S\,C=\frac{2\,T\,P}{2\,T\,P+F\,P+F\,N},S\,E\,N=\frac{T\,P}{T\,P+F\,N},P\,P\,V=\frac{T\,P}{T\,P+F\,P}$$

Where TP, FP, and FN denote the true positive, false positive, and false negative, respectively. DSC reflects the overall segmentation performance, SEN indicates the capability of detecting the PVSs voxels, and PPV represents the capability of discarding the confounding background voxels. The correlation of PVSs count between the model segmentation results and the original T2-weighted images was obtained by calculating the Spearman’s correlation coefficient.

## 3 Results

### 3.1 Segmentation performance

**FIGURE 3:**
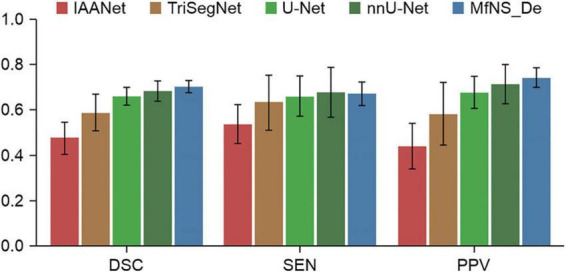
Comparison of segmentation results from five different models.

Table 2 and Figure 3 present the segmentation results obtained using our MfNS_De method and the four other comparison methods (IAANet, TriSegNet, U-Net, and nnU-Net). We could obtain the following observations: first, our method achieved approximately 23%, 11%, 4%, and 2% average DSC enhancement, respectively. Second, the proposed MfNS_De outperformed the original U-Net mainly because of the use of two key modules in the proposed method: the densely nested U-Net structure and the multi-scale feature learning strategy supervised by multi-scale down sampling of the label images.

**TABLE 2:** Segmentation results obtained by five different models: the best scores are highlighted as boldface.

Subject	DSC					SEN					PPV				
	IAANet	TriSeg Net	U-Net	nnU-Net	MfNS _De	IAANet	TriSeg Net	U-Net	nnU-Net	MfNS _De	IAANet	TriSeg Net	U-Net	nnU-Net	MfNS _De
1	0.492	0.643	0.694	**0.722**	0.714	0.577	0.689	0.666	**0.802**	0.631	0.429	0.603	0.724	0.656	**0.821**
2	0.510	0.596	0.720	0.687	**0.737**	0.661	0.492	0.673	0.609	**0.755**	0.415	0.755	0.774	**0.788**	0.717
3	0.458	0.533	0.676	0.678	**0.703**	0.435	**0.843**	0.804	0.838	0.633	0.483	0.390	0.583	0.570	**0.793**
4	0.431	0.574	0.640	0.677	**0.685**	0.519	0.710	0.617	**0.816**	0.630	0.369	0.482	0.664	0.579	**0.717**
5	0.498	0.662	0.646	0.652	**0.675**	0.432	**0.669**	0.535	0.522	0.649	0.587	0.654	0.816	**0.869**	0.697
6	0.412	0.555	0.642	0.668	**0.718**	0.445	0.780	0.627	0.614	**0.786**	0.383	0.431	0.658	**0.732**	0.665
7	0.491	0.611	0.636	0.630	**0.683**	0.442	0.580	0.598	0.548	**0.679**	0.551	0.645	0.680	**0.741**	0.682
8	0.409	0.313	0.629	0.651	**0.671**	0.431	0.535	0.645	0.559	**0.651**	0.389	0.221	0.613	**0.779**	0.701
9	0.561	0.647	0.732	**0.753**	0.711	0.493	0.544	0.689	**0.693**	0.630	0.651	0.797	0.781	**0.824**	0.803
10	0.617	0.582	0.649	0.676	**0.703**	0.729	0.605	0.662	0.737	**0.750**	0.535	0.561	0.637	0.624	**0.740**
11	0.600	0.500	0.580	0.630	**0.699**	0.636	0.376	0.472	0.527	**0.645**	0.567	0.745	0.752	**0.785**	0.775
12	0.581	0.538	0.581	0.591	**0.675**	0.613	0.452	0.488	0.486	**0.634**	0.552	0.665	0.719	**0.752**	0.735
13	0.445	0.598	0.659	**0.690**	0.663	0.541	0.622	0.643	**0.667**	0.600	0.378	0.575	0.677	0.714	**0.726**
14	0.374	0.568	0.649	**0.715**	0.687	0.460	0.744	0.709	**0.763**	0.659	0.315	0.459	0.597	0.674	**0.726**
15	0.411	0.576	0.646	0.667	**0.691**	0.562	0.563	0.717	**0.717**	0.678	0.323	0.589	0.589	0.624	**0.702**
16	0.474	0.673	0.700	**0.776**	0.745	0.621	0.792	0.744	**0.822**	0.706	0.383	0.585	0.661	0.735	**0.770**
17	0.431	0.681	0.706	0.733	**0.750**	0.525	0.655	0.715	**0.716**	0.706	0.365	0.709	0.696	0.750	**0.799**
18	0.425	0.654	0.665	0.695	**0.702**	0.542	0.697	0.723	**0.796**	0.675	0.350	0.616	0.616	0.617	**0.733**
19	0.484	0.674	0.715	0.710	**0.744**	0.535	0.696	0.732	0.643	**0.738**	0.441	0.654	0.699	**0.792**	0.751
20	0.439	0.608	0.649	0.666	**0.686**	0.551	0.656	**0.742**	0.668	0.618	0.365	0.567	0.576	0.664	**0.769**
Mean	0.477	0.589	0.661	0.683	**0.702**	0.537	0.635	0.660	**0.677**	0.673	0.442	0.585	0.676	0.713	**0.741**
Std	0.066	0.081	0.040	0.043	**0.025**	0.082	0.116	0.084	0.108	**0.050**	0.096	0.134	0.068	0.082	**0.042**

In the three evaluation metrics (DSC, SEN, PPV), bold values indicate the highest corresponding evaluation scores for the models.

**FIGURE 4:**
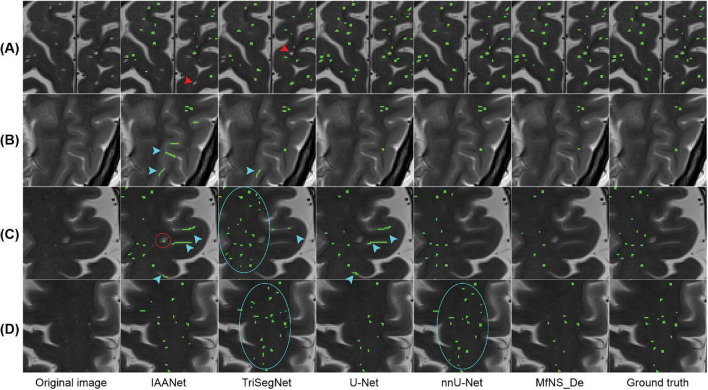
Illustration of typical PVSs segmentation by five different models. **(A–D)** Indicate different subjects, respectively. Red arrows indicate PVSs that are not successfully segmented; blue arrows indicate the identification of the sulcus as PVSs; red circle indicates lacunar infarction; and blue circles indicate the identification of some of these slightly higher signaling voxels as PVSs. PVSs, perivascular spaces.

Figure 4 shows the segmentation results for several typical PVSs. Clearly, IAANet and TriSegNet could not detect many PVSs of normal signals (Figure 4A). Additionally, IAANet, TriSegNet, and U-Net faced difficulty in distinguishing some of the fine sulci from the PVSs (Figures 4B, C), resulting in many sulci being incorrectly categorized as PVSs. Moreover, the IAANet was unable to distinguish some lacunar infarctions (Figure 4C). Similarly, TriSegNet and nnU-Net were prone to detecting some slightly higher-signal non-PVS voxels as PVSs (Figures 4C, D), leading to a large number of misreported voxels. Notably, MfNS_De effectively resolves the above problems, and its segmentation results are more consistent with the ground truth.

**FIGURE 5:**
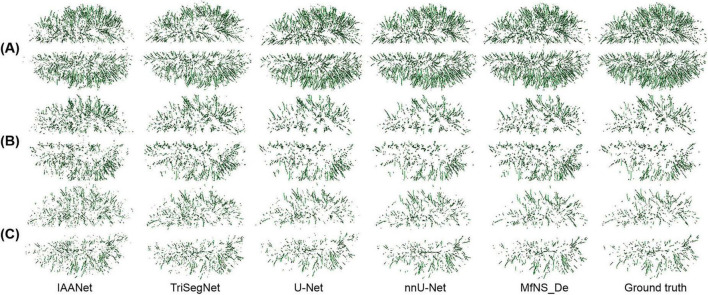
3D results of the segmentation of large amounts of PVSs **(A)**, medium amounts of PVSs **(B)**, and small amounts of PVSs **(C)** using five different models, shown in axial view. PVSs, perivascular spaces.

Figure 5 shows a 3D view of different PVSs burdens, illustrating the distribution of whole-brain PVSs. In Figures 5B, C, IAANet and TriSegNet demonstrate a significantly higher whole-brain PVSs burden than the ground truth, and the PVSs morphology appears more irregular, which is consistent with the results shown in Figure 4. In contrast, the 3D view demonstrated by MfNS_De and nnU-Net closely resembles the ground truth. However, in Figure 5B, we can see that MfNS_De performs better than nnU-Net for detecting PVSs at the brain’s edges.

### 3.2 Visual count comparison

**FIGURE 6:**
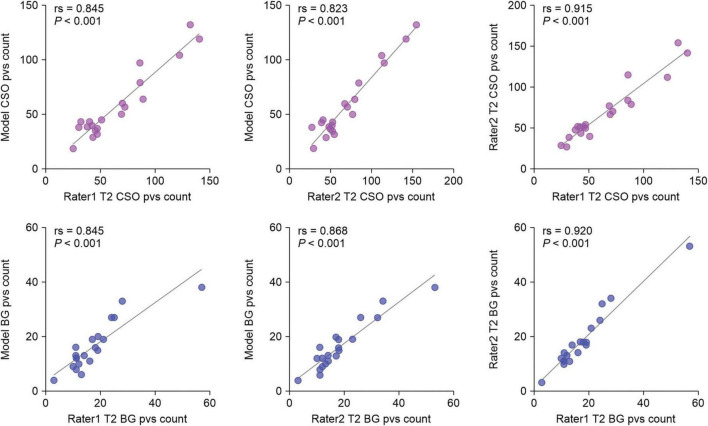
Scatter plots of visual counts of the PVSs between T2-weighted images and our MfNS_De segmentation results. Purple and blue denote centers of semiovales (CSO) and basal ganglia (BG), respectively; rs denotes Spearman’s correlation coefficient.

We evaluated the ability of our MfNS_De model to detect PVSs by comparing them with expert visual counts. Figure 6 shows the scatter plots and Spearman’s correlation coefficients for PVSs counts in the BG and CSO. There was a very high correlation between counts by visual raters and our model’s detection of PVSs in the same section (BG:rs = 0.845, *P* < 0.001; rs = 0.868, *P* < 0.001; CSO:rs = 0.845, *P* < 0.001; rs = 0.823, *P* < 0.001 for raters 1 and 2, respectively), and this result almost reached an inter-rater correlation (rs = 0.920, *P* < 0.001; rs = 0.915, *P* < 0.001 for BG and CSO, respectively). Overall, the PVSs count from our model’s automatic segmentation was lower than that from T2-weighted MRI, which could be because the model ignored very small and low-signal PVSs. The data collected had a heavy PVSs burden, with counts reaching 132 (CSO) and 38 (BG) based on our model’s segmentation results. As shown in Figure 6, one can notice that even for scans with a heavy PVSs burden, the PVSs counts from the automatic segmentation of the model were satisfactory. If humans were to visually rate scans with many PVSs on the original T2-weighted images alone, it would be a very time-consuming and labor-intensive task, and the results might also be more variable owing to subjective judgment.

## 4 Discussion

An increasing number of studies have indicated that PVSs are associated with cerebrospinal fluid circulation, clearance of metabolic waste from the brain, and certain neurodegenerative diseases (Iliff et al., 2013; Rangroo Thrane et al., 2013; Yang et al., 2013; Francis et al., 2019). Accurate and convenient evaluation of PVSs has been widely discussed in recent years. In this study, we proposed an automatic PVSs segmentation method of the whole brain in T2-weighted 3T MRI based on deep learning and achieved excellent segmentation results. We compared the segmentation accuracy between the MfNS_De method and the other four methods (IAANet, TriSegNet, U-Net, and nnU-Net). Our method achieved about 23%, 11%, 4%, and 2% average DSC improvement, respectively.

nnU-Net utilizes the original U-Net structure to optimize segmentation results through pre-processing and post-processing. It achieved the best automatic segmentation performance on 33 of the 53 anatomical structures evaluated, demonstrating strong generalization characteristics. nnU-Net does not require expert knowledge or computational resources beyond standard network training, and no manual task-specific adaptation is necessary, which is why we chose nnU-Net for comparison. Our results showed that nnU-Net exhibited higher sensitivity than all the other models, including MfNS_De, but at the cost of a lower PPV. In other words, nnU-Net accurately detects PVS but also includes more false positive regions, which can be clinically harmful. Therefore, we still consider DSC to be a more reasonable metric. It is noteworthy that nnU-Net’s DSC of 0.68 does not reach MfNS_De’s value of 0.702, indicating that we outperform nnU-Net in the PVS segmentation task.

The nested design of our network architecture allows multi-scale feature learning, which is crucial in image segmentation tasks, where objects of interest vary in size and shape. This network can effectively capture features at different scales, thereby improving its accuracy and robustness in segmenting small objects. The densely nested U-Net architecture enhances feature fusion from different layers. By combining features across various depths of the network, the model can leverage both low-level texture and edge details, as well as high-level contextual information. This comprehensive feature integration is particularly effective in achieving precise and detailed segmentation results, making it a powerful tool for various image segmentation applications. Moreover, multi-scale HFs facilitate the detection and segmentation of small objects by focusing on features at various scales. This is particularly useful for capturing the nuances of smaller objects that may be lost on a single scale or using a conventional U-Net model.

As shown in Figures 4, 5, IAANet and TriSegNet either frequently detect many anomalous voxels as PVSs or miss many true PVSs. The U-Net cannot distinguish between a small number of blended boundaries. Similarly, nnU-Net tends to detect many slightly higher-signal non-PVS voxels as PVSs, which results in nnU-Net’s SEN scores being slightly higher than those in our method; however, its DSC and PPV scores were lower than those in our method owing to a higher false positive rate. Our method clearly outperforms these four comparison models. Specifically, we proposed the MfNS_De model, which introduces a novel approach to image segmentation that is particularly effective for small objects, such as PVS. Its unique architectural feature, namely densely nested layers, facilitates a comprehensive understanding of both high-level and low-level image details, which is crucial for accurately segmenting small objects. The integration of features from different network depths yields precise and detailed segmentation results. Additionally, deep supervision with multi-scale HFs introduces a novel approach for segmenting small objects, combining the strengths of multiple layers of models with the precision of multi-scale analysis. The integration of multi-scale HFs allows the effective capture of detailed features at various scales, which is crucial for the precise identification of smaller objects that are often missed when using traditional methods. This combination significantly reduces false positives and improves segmentation accuracy. Overall, the proposed MfNS_De model effectively improves the segmentation accuracy by integrating different network depth features as well as multi-scale salient foreground depth supervision methods. The segmentation results show that the proposed strategy improves the segmentation performance, and the MfNS_De method detects PVSs in the whole brain so that a 3D view of the PVSs can be obtained (Figure 5), which helps doctors visualize the morphology, number, and distribution of PVSs in the brain.

Numerous studies have investigated the automatic segmentation of PVSs (Hou et al., 2017; Zhang et al., 2017; Lian et al., 2018; Boutinaud et al., 2021; Spijkerman et al., 2022; Rashid et al., 2023). Lian et al. (2018) used a fully convolutional neural network (FCNN) machine learning approach on a dataset of T2-weighted magnetic MRI acquired using a 7 T scanner. The DSC of their method was 0.77, which is the highest result among the articles we retrieved thus far. Given that 7TMR imaging is rarely used in clinical practice, the segmentation of PVSs in 3T scanners is beneficial for studying various diseases. However, the number of current research on automatic PVSs segmentation in 3T scanners is limited (Boutinaud et al., 2021; Rashid et al., 2023); in the literature we have retrieved, the reported DSC has not exceeded 0.70, except for some specific PVS (PVS clusters and large PVSs) (Boutinaud et al., 2021). Most articles have not disclosed the dice score, a measure of overall segmentation performance and the most rigorous metric for evaluating the performance of PVSs segmentation algorithms (Pham et al., 2022). This also reinforces the clinical value of our 3T MRI model.

We found some literature that showcases several excellent methods for brain tumor segmentation. Liu et al. (2022) presented a glioma segmentation method based on adversarial learning. It fuses contrast-enhanced T1-weighted and Flair MRI images, using a semantic segmentation network as a discriminator to extract tumor-related information. Zhu et al. (2023) proposed a brain tumor segmentation method based on the fusion of deep semantics and edge information. It primarily utilizes an improved Swin Transformer for semantic segmentation, combines convolutional neural network for edge detection, and employs graph convolution for feature fusion. The method aims to enhance segmentation accuracy using multimodal MRI data by fully leveraging deep semantic features and edge features. Afterward, Zhu et al. (2024) proposed a new brain tumor segmentation method that consists of three modules, the Modality Information Extraction Module (MIE) for weighting different modality information, the Spatial Information Enhancement Module (SIE) for enhancing spatial information extraction through dilated convolutions, and the Boundary Shape Correction Module (BSC) for improving segmentation accuracy by selecting and constraining critical boundary points. I believe our upcoming PVS segmentation work can draw new ideas from these articles to build a more efficient PVS segmentation model.

**FIGURE 7:**
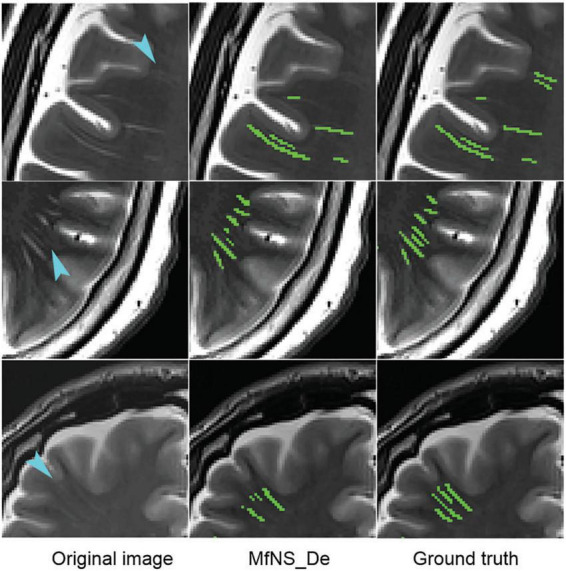
Typical cases of MfNS_De method segmentation failure. Blue arrows indicate low-signal PVSs. PVSs, perivascular spaces.

Our study has some limitations. First, as shown in Figure 7, failed segmentation may occur in a few cases. The MfNS_De method may not detect PVSs with very low signals on T2-weighted images, leading to a lack of continuity in the segmentation results for long PVSs with uneven signal strengths along the extension direction. Moreover, a possible impact to model generalizability since the images were from PD patients. For future work, we need to consider a large sample to improve the model’s generalizability, including children, normal persons, and patients with other diseases. Finally, high-resolution T2 (slice thickness: 2 mm/slice gap: 0 mm) can reduce motion artifacts and display the anatomical structures of the BG more clearly (Rasouli et al., 2018; Vos et al., 2018). Therefore, we set the slice thickness of the T2-weighted images to 2mm, but this may also cause us to miss some unscanned PVSs.

## 5 Conclusion

In this study, we introduced a multi-scale supervised dense nested attention network designed to segment the PVS based on T2-weighted 3T MRI. Our method achieved the best segmentation results compared with the four other deep-learning-based methods, reaching a DSC of 0.702. The results of the visual count of PVSs in our model showed an extremely high correlation with those of experts on T2-weighted images (BG: rs = 0.845, *P* < 0.001; rs = 0.868, *P* < 0.001; CSO: rs = 0.845, *P* < 0.001; rs = 0.823, *P* < 0.001 for raters 1 and 2, respectively). We believe that this method will facilitate practical clinical applications and is expected to replace the method of direct visual counting directly on T1-weighted images or T2-weighted images.

## Data Availability

The original contributions presented in the study are included in the article/supplementary material, further inquiries can be directed to the corresponding authors.
